# Supplementary material for: Integrated analysis of Xist upregulation and X-chromosome inactivation with single-cell and single-allele resolution
Source: Nat Commun. 2021 Jun 15;12:3638. doi: 10.1038/s41467-021-23643-6 (PMC8206119; doi:10.1038/s41467-021-23643-6)
Supplement: Supplementary file 1 — Supplementary Information [file 41467_2021_23643_MOESM1_ESM.pdf]

Integrated analysis of Xist upregulation and X-chromosome inactivation with single-cell and single-allele resolution

Guido Pacini, Ilona Dunkel, Norbert Mages, Verena Mutzel, Bernd Timmermann, Annalisa Marsico, Edda G Schulz

Supplementary Information

Supplementary Figure 1

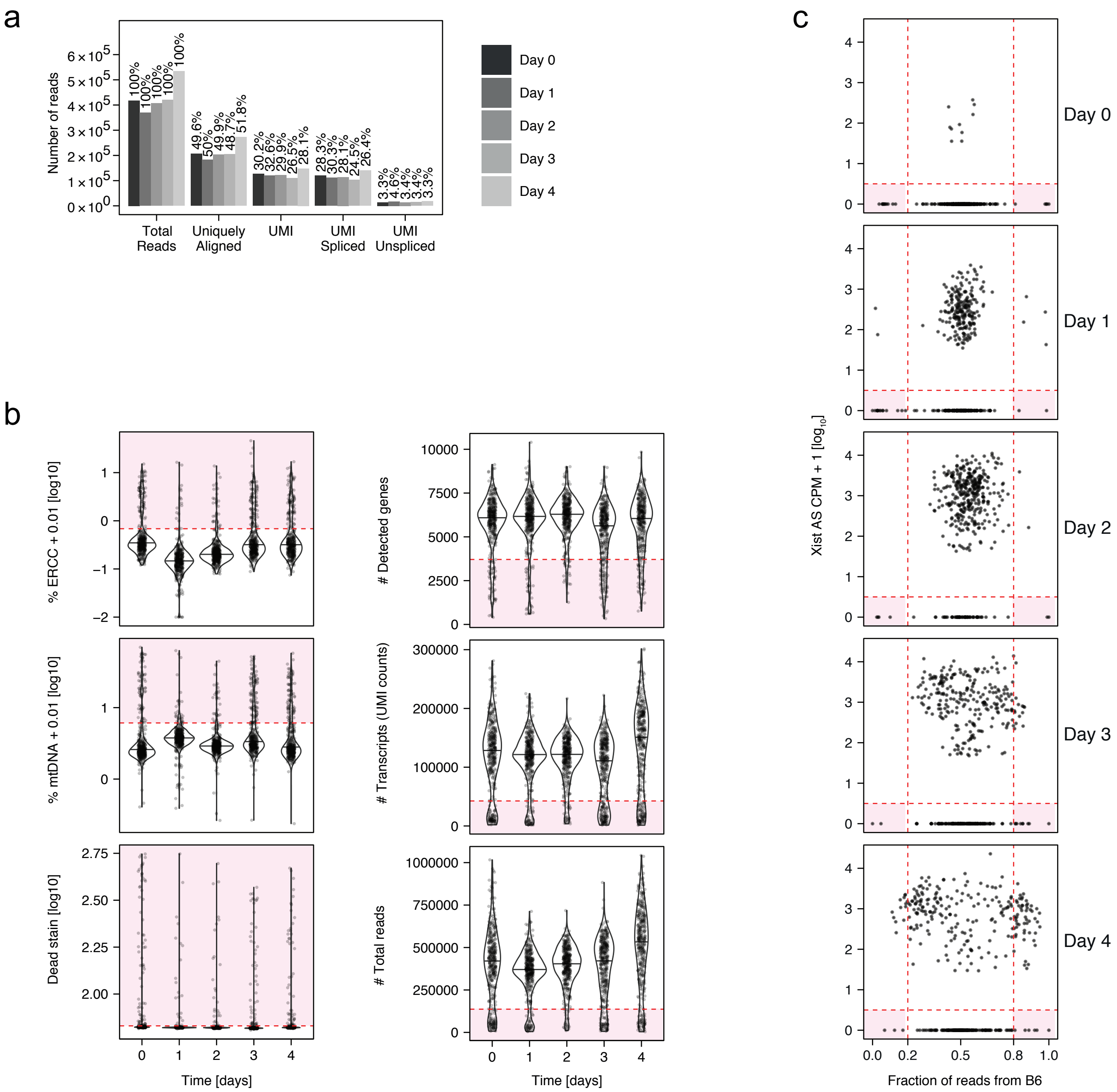

Supplementary Figure 1. Mapping statistics and data pre-processing

(a) Alignment statistics, showing the median number of reads across all sequenced cells (n=400 cells per time point) for the indicated read groups. **(b)** Violin plots of the parameters used for cell filtering. Cells falling in the shaded areas were excluded from the analyses. The horizontal lines indicate the median value. **(c)** Putative XO cells were identified as AS Xist-negative cells, where >80% of X-chromosomal reads mapped to the same allele (shaded areas), and were excluded from the analyses. Source data are provided as a Source Data file.

Supplementary Figure 2

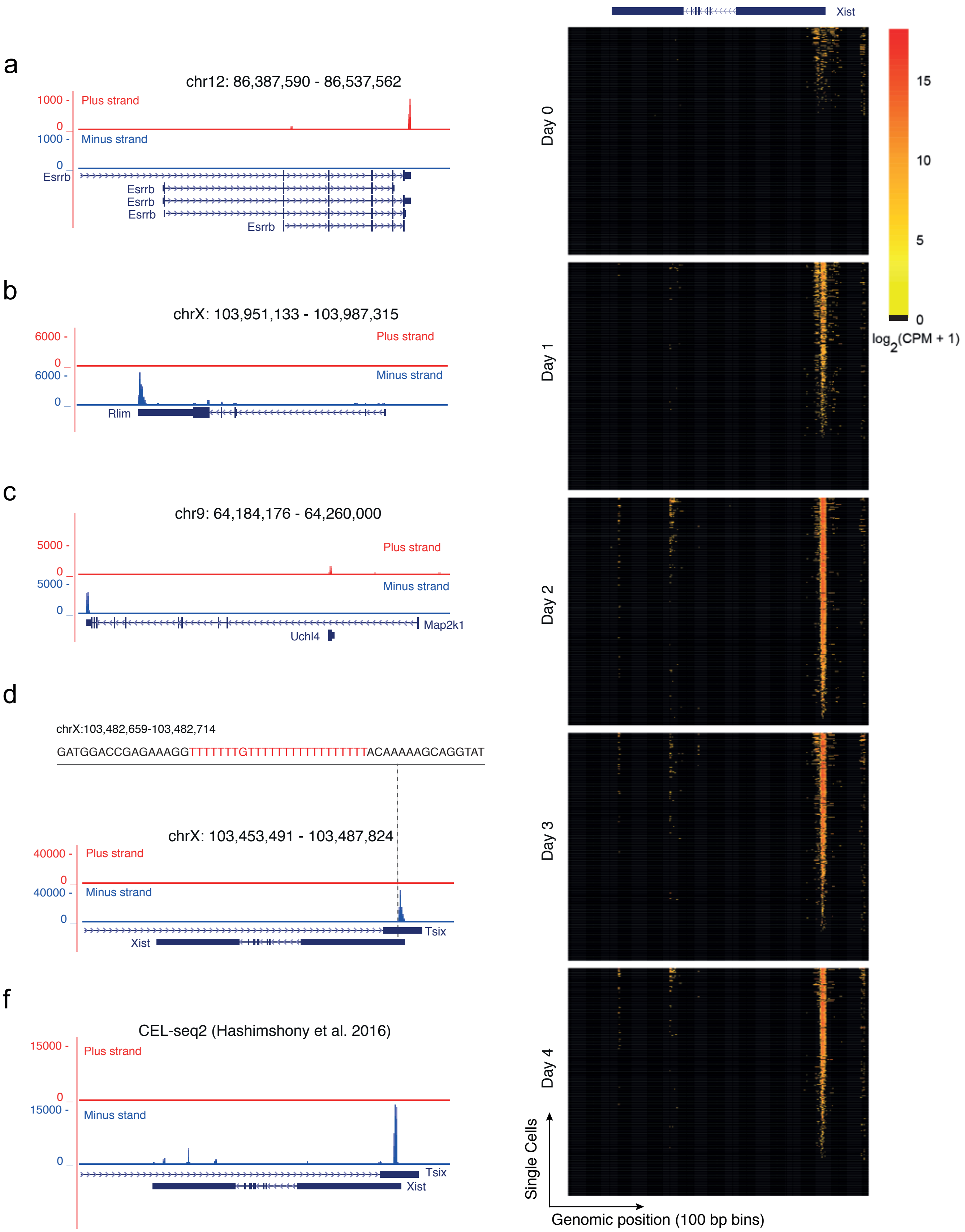

Supplementary Figure 2: Unusual alignment pattern across the *Xist* gene

(a-d) Composite tracks across all cells show read distributions for three representative control genes (a-c) and Xist (d). In (d) a sequence segment next to the region to which most reads map is shown in detail. A polyT stretch that will generate a genomically encoded polyA stretch in the Xist RNA appeared to prime the reverse transcription reaction during library preparation. **(e)** Heatmaps describing the 100bp-binned read coverage across the *Xist* gene on the minus strand (chrX: 103,460,366 - 103,483,254, mm10) for all cells (rows), separately ordered for each time point by the total number of reads mapping to Xist. **(f)** Composite track around *Xist* as in (d), for a previously published scRNA-seq data set of murine fibroblasts using the CEL-seq2 protocol[1].

Supplementary Figure 3

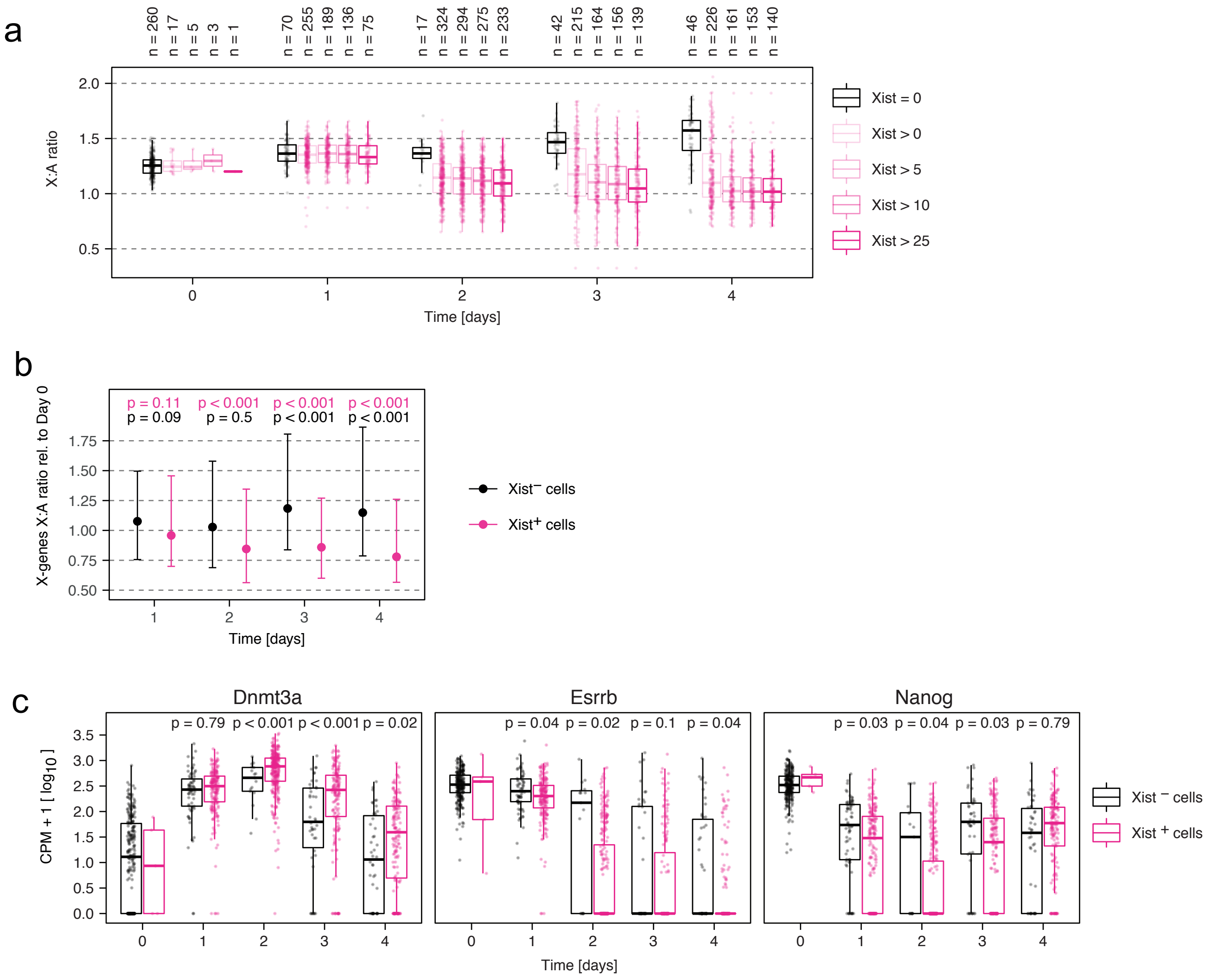

Supplementary Figure 3. Marker gene expression and X:A expression ratios

(a) Box plot of the X-to-autosome expression ratio in Xist-positive (pink) and Xist-negative cells (black) for different count thresholds to classify cells as Xist-positive (0, 5, 10, 25). The number of cells in each group is given on top. **(b)** Average expression of each X-linked gene (n=372) in Xist+ and Xist- cells relative to average autosomal gene expression was normalized to the respective value at day 0. Autosomal genes not expressed at day 0 were excluded from the analysis. The median (circles) and the 25th and 75th percentiles (whiskers) are shown. Additionally, p-values of a Sign two-sided test (H0: median = 1) are shown to test for a significant difference compared to day 0. **(c)** Box plots showing the distribution of the normalized expression of 3 marker genes in Xist-positive and Xist-negative cells. Additionally, p-values comparing the two groups with a two-sample unpaired two-sided Student's T-test are shown. In (a) and (c) the central mark indicates the median, and the bottom and top edges of the box indicate the first and third quartiles, respectively. The top and bottom whiskers extend the boxes to a maximum of 1.5 times the interquartile range. Dots represent individual cells. Source data are provided as a Source Data file.

Supplementary Figure 4

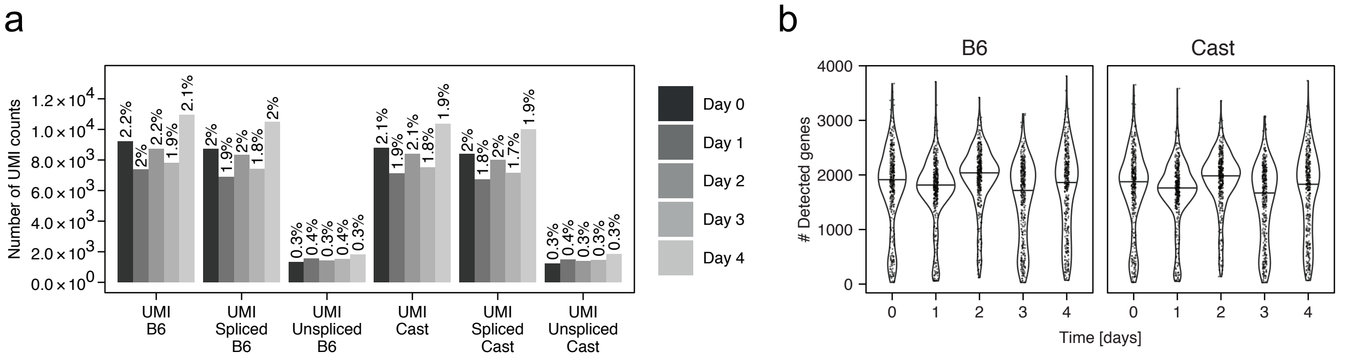

Supplementary Figure 4. Allele-specific mapping statistics

(a) Alignment statistics, showing the median number of allele-specific reads across all sequenced cells (n=400 cells per time point) for the indicated read groups. **(b)** Violin plot showing the distribution of the number of detected genes (UMI > 0) with allelic resolution. Dots represent individual cells, horizontal lines indicate the median values. Source data are provided as a Source Data file.

Supplementary Figure 5

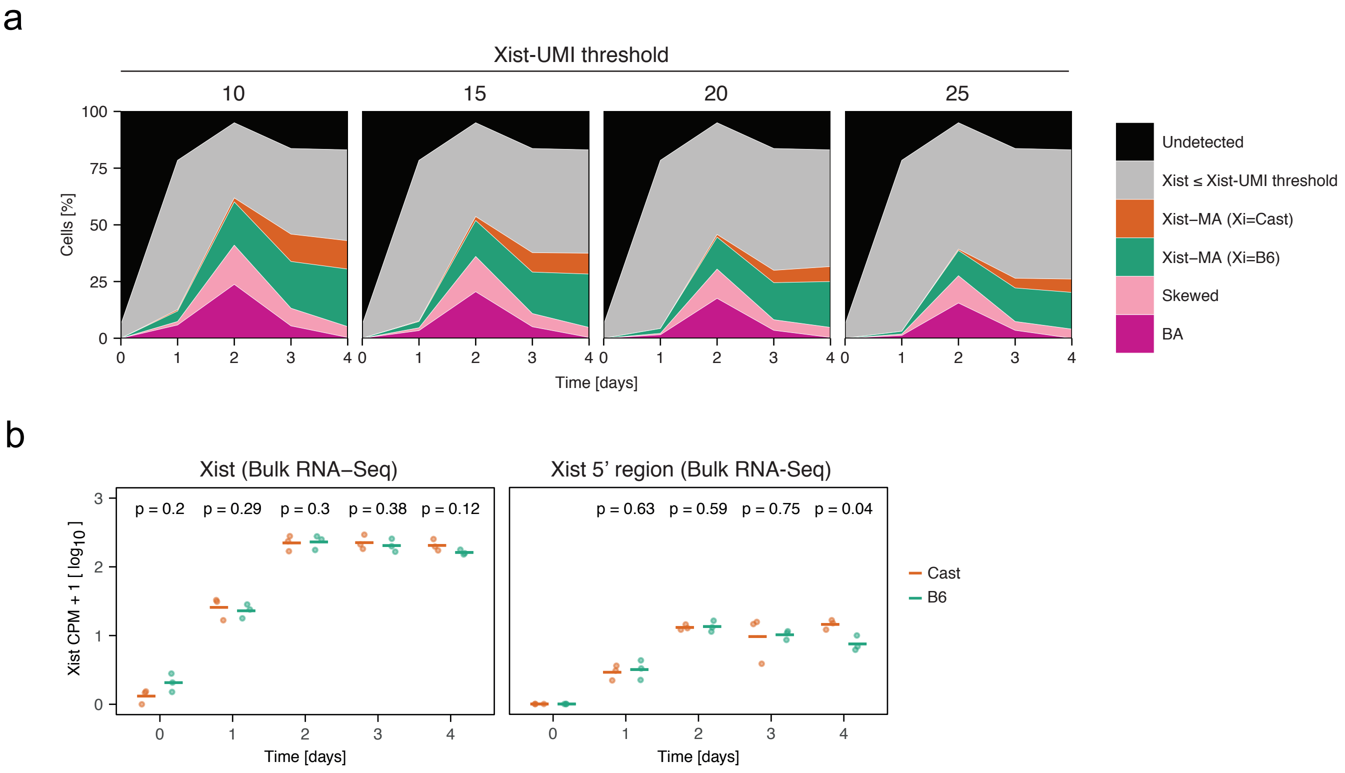

Supplementary Figure 5. Xist classification and allelic expression analysis

(a) Xist pattern classification as in main Fig. 2b for different Xist-UMI threshold values, above which cell classification according to the allelic Xist expression pattern was performed (MA, BA, Skewed). **(b)** Allele-specific Xist expression levels estimated from bulk RNA-seq data in the TX1072 cell line, using all Xist SNPs (left), or only two SNP in Xist's 5'-end (right, chrX: 103,482,240 and chrX: 103,482,895, mm10) that were used for the scRNA-seq analysis. Horizontal lines indicate the mean across n=3 biological replicates (dots). Additionally, p-values of a two-sample two-sided paired Student's T-test are shown. Source data are provided as a Source Data file.

Supplementary Figure 6

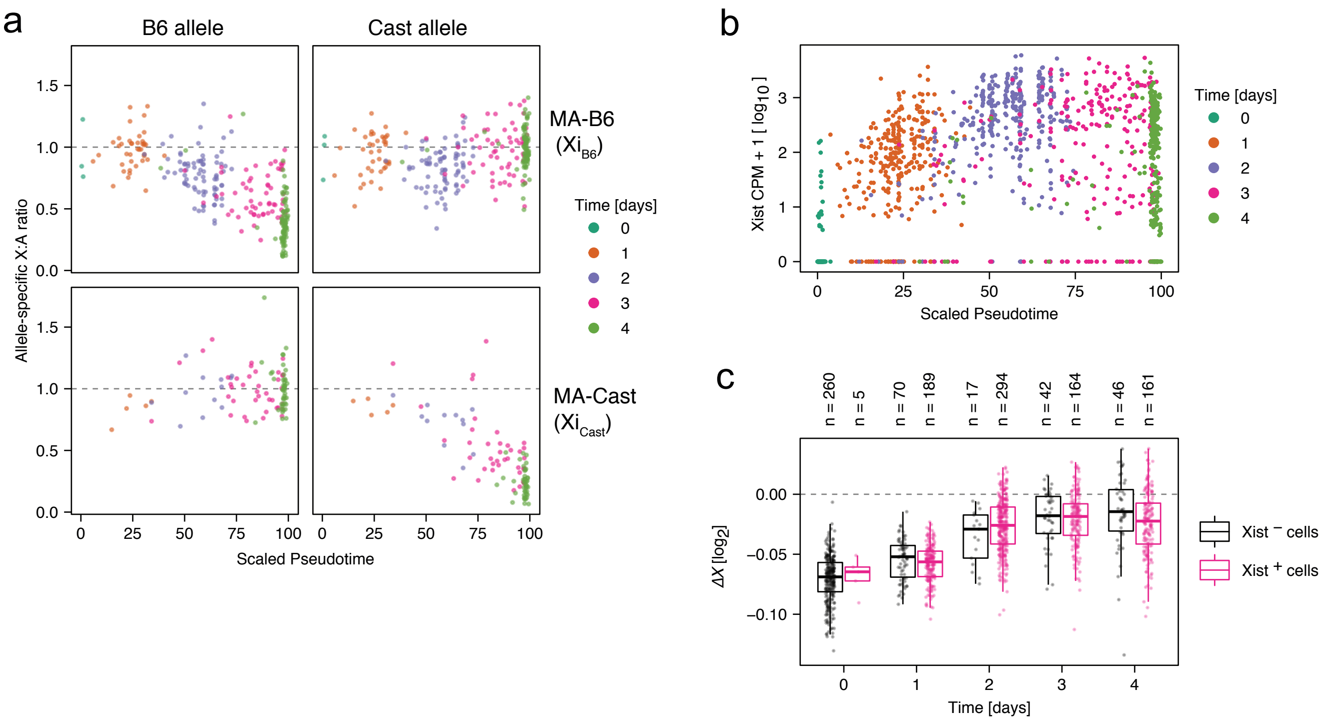

Supplementary Figure 6. XCI and Xist expression is variable across pseudotime

(a) Comparison of allele-specific X-to-Autosome ratios for B6 (left) and Cast chromosomes (right) with scaled pseudotime as in Fig. 1b for MA-B6 (top) and MA-Cast cells (bottom). Cells are colored according to measurement time point. **(b)** Comparison of Xist expression levels and scaled pseudotime as in Fig. 1b. **(c)** Box plot of the log-ratio between RNA-velocity estimates of current and predicted normalized X-chromosomal gene expression (ΔX) in Xist-positive (pink) and Xist-negative cells (black). The number of cells in each group is given on top. The central mark indicates the median, and the bottom and top edges of the box indicate the first and third quartiles, respectively. The top and bottom whiskers extend the boxes to a maximum of 1.5 times the interquartile range. Dots represent individual cells. Source data are provided as a Source Data file.

Supplementary Figure 7

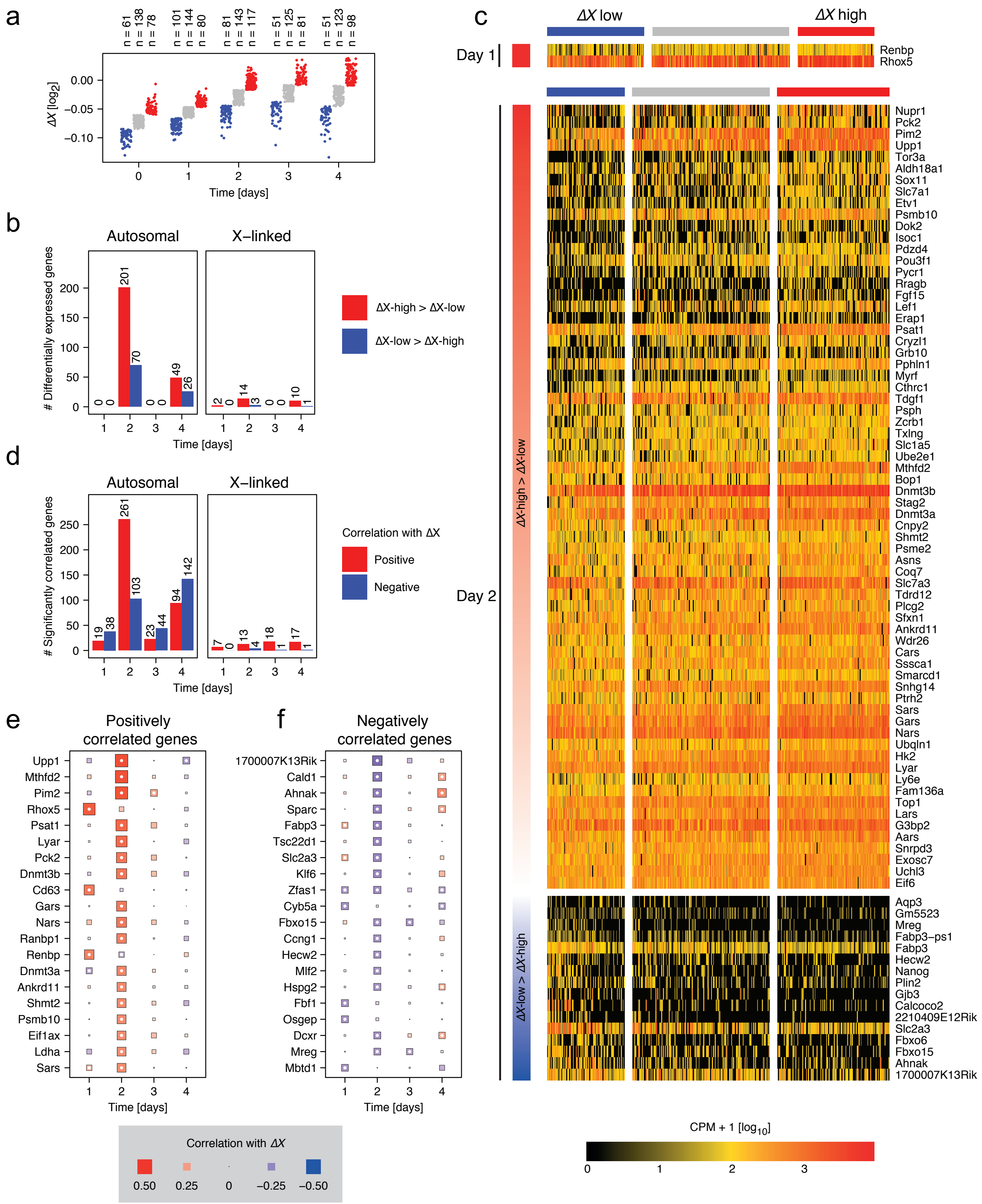

Supplementary Figure 7: Identification of putative XCI regulators through velocity-based Δ*X* analysis

(a) Cell classification according to log-ratio between RNA-velocity estimates of current and predicted normalized X chromosome gene expression (Δ*X*). The highest and lowest cell clusters from a K-means clustering (*K*=3) were classified as Δ*X*-high (red) and Δ*X*-low (blue). **(b)** Number of differentially expressed genes (DEGs), excluding Xist, between Δ*X*-high and Δ*X*-low cells on autosomes (left) and on the X chromosome (right) (BH-corrected p-value ≤ 0.05). **(c)** Heatmaps showing expression across cells for genes with BH-corrected p-value ≤ 0.01 with absolute fold change between Δ*X*-high and Δ*X*-low cells above 1.5 at day 1 (top) or day 2 (bottom). Cells are grouped according to the clustering shown in (a), genes are ordered by decreasing fold change. X-linked genes with Δ*X*-low>Δ*X*-high are not shown. **(d)** Number of genes, whose expression is positively (red) or negatively (blue) correlated with the Δ*X* estimate (Spearman's correlation test and BH corrected p-value ≤ 0.05) across cells of the same time point. **(e-f)** Spearman's correlation coefficients with the Δ*X* estimate for positively (e) and (autosomal) negatively (f) correlated genes, excluding pseudogenes. Top 20 genes that exhibit a significant correlation (Spearman's correlation test and BH-corrected p-value ≤ 0.05) at day 1 or 2 are shown, ordered by decreasing absolute correlation coefficient. Size and color indicate the correlation coefficient as indicated. White dots represent significant correlations. Source data are provided as a Source Data file.

Supplementary Figure 8

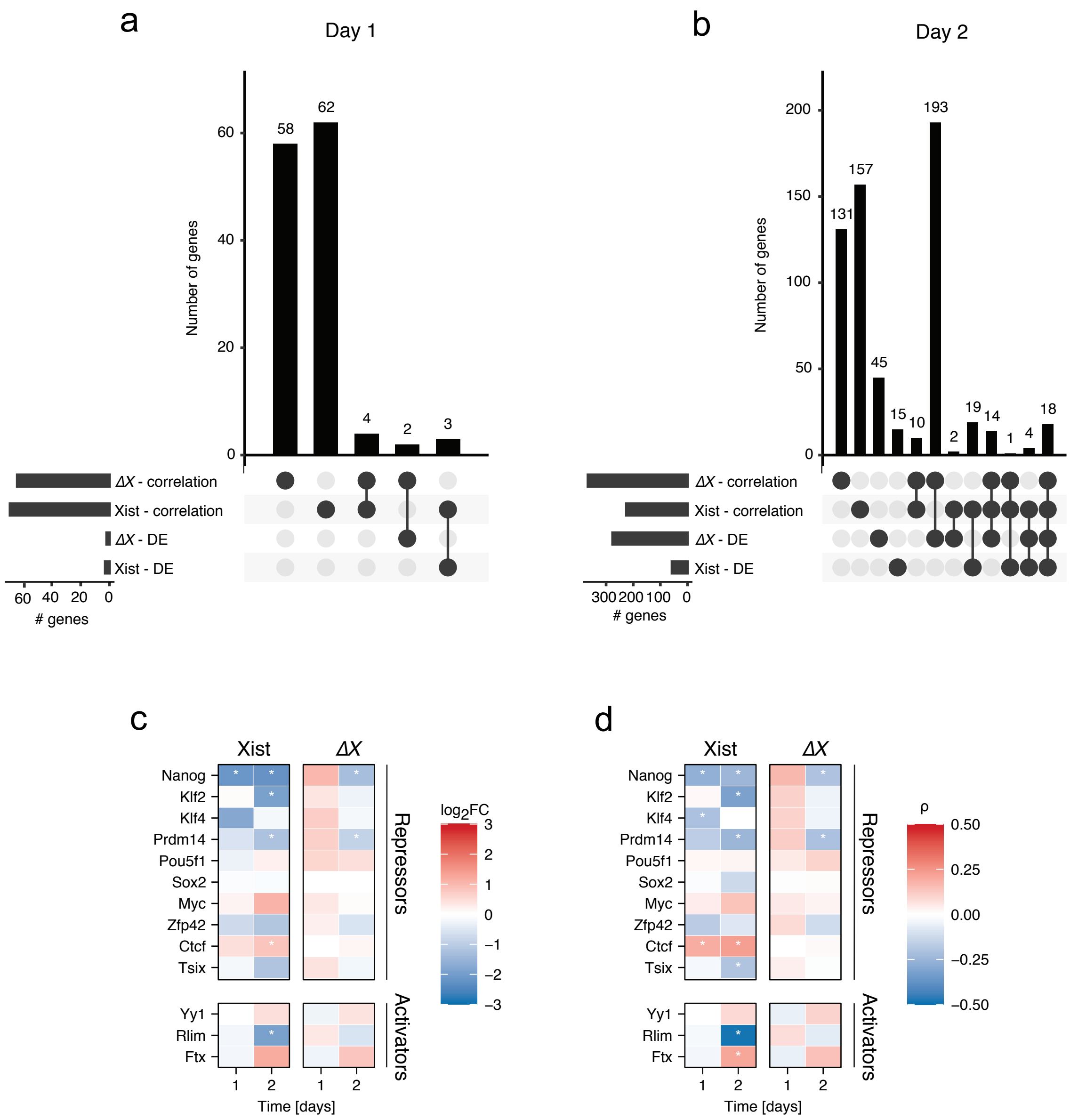

Supplementary Figure 8: Identification of putative XCI regulators

(a-b) Overlap between putative regulators of early XCI identified through correlation and differential expression analyses (BH-corrected p-value ≤ 0.05) based on Xist expression and early gene silencing (Δ*X*) at day 1 (a) and 2 (b) of differentiation, excluding pseudogenes and X-linked genes with a negative correlation coefficient or $\log_2$FC (8501 autosomal and 346 X-linked genes tested). **(c-d)** The $\log_2$-transformed fold changes $\log_2$FC in (c) and Spearman's correlation coefficient in (d) are shown as in Fig. 5b+c for previously proposed Xist regulators, all of which, except Jpx, could be efficiently detected in the data set. Asterisks indicate significant genes (BH-corrected p-value ≤ 0.05). Source data are provided as a Source Data file.

Supplementary Figure 9

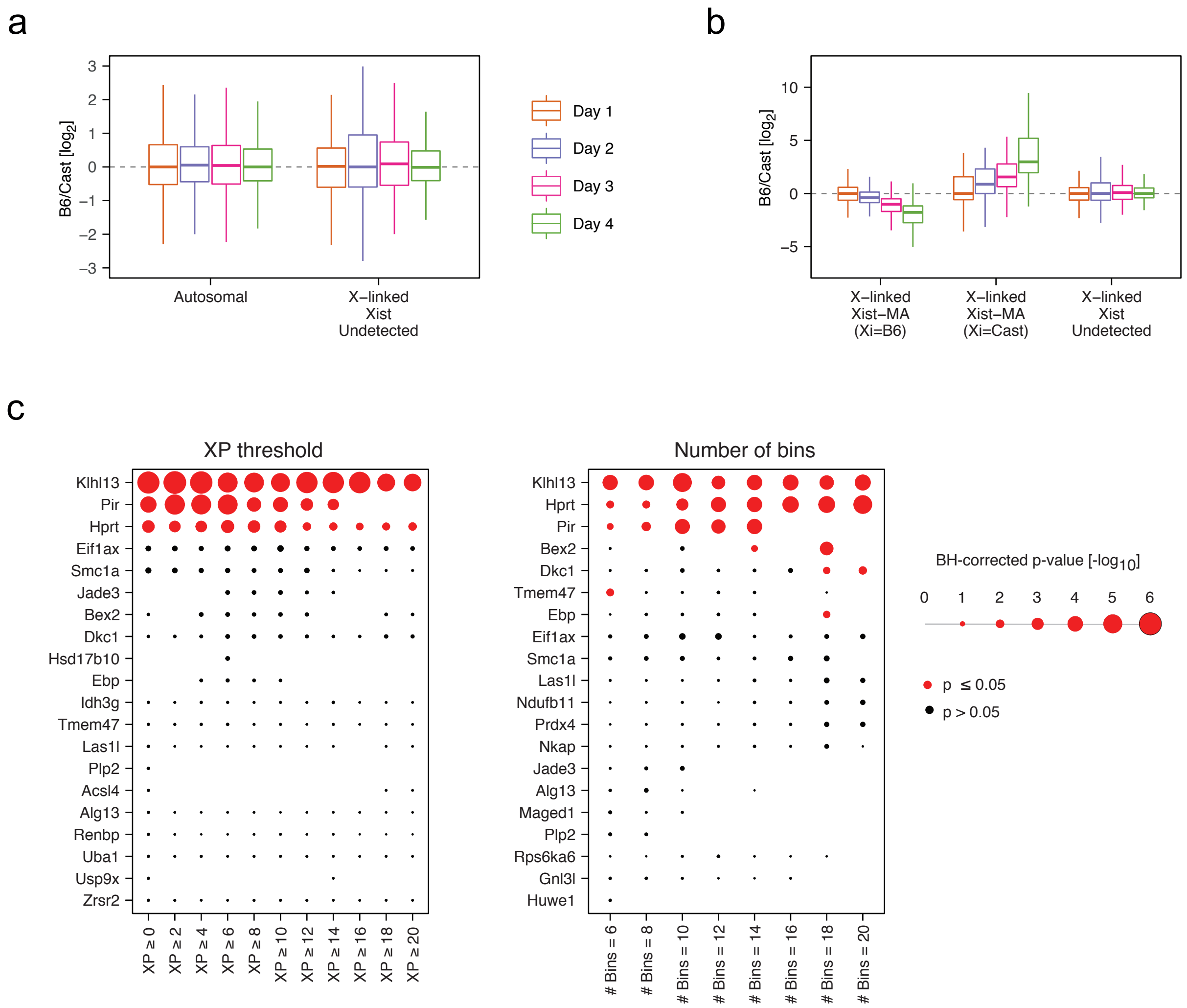

Supplementary Figure 9: Gene Silencing analysis

(a-b) Box plots showing the distribution of the B6-to-Cast ratio of all autosomal (n=4859) and X-linked genes (n=159) as indicated, calculated by pooling all cells (for autosomal genes) and the indicated cell subsets for X-linked genes. The central mark indicates the median, and the bottom and top edges of the box indicate the first and third quartiles, respectively. The top and bottom whiskers extend the boxes to a maximum of 1.5 times the interquartile range. Outliers are not shown. **(c)** The differential silencing analysis in Fig. 6 was repeated with different values for the analysis parameters. The minimum XP threshold used to filter the cells included in the analysis (left) and number of bins into which the cells were grouped (right) were varied. The dot plot shows the results for the 20 genes (rows) resulting in the smallest BH-corrected p-values across all parameter sets (columns). The analysis was not performed (missing dot) for those parameter sets resulting in less than 5 bins containing a minimum of 5 cells or less than 25 total allele-specific counts. Source data are provided as a Source Data file.

Supplementary Figure 10

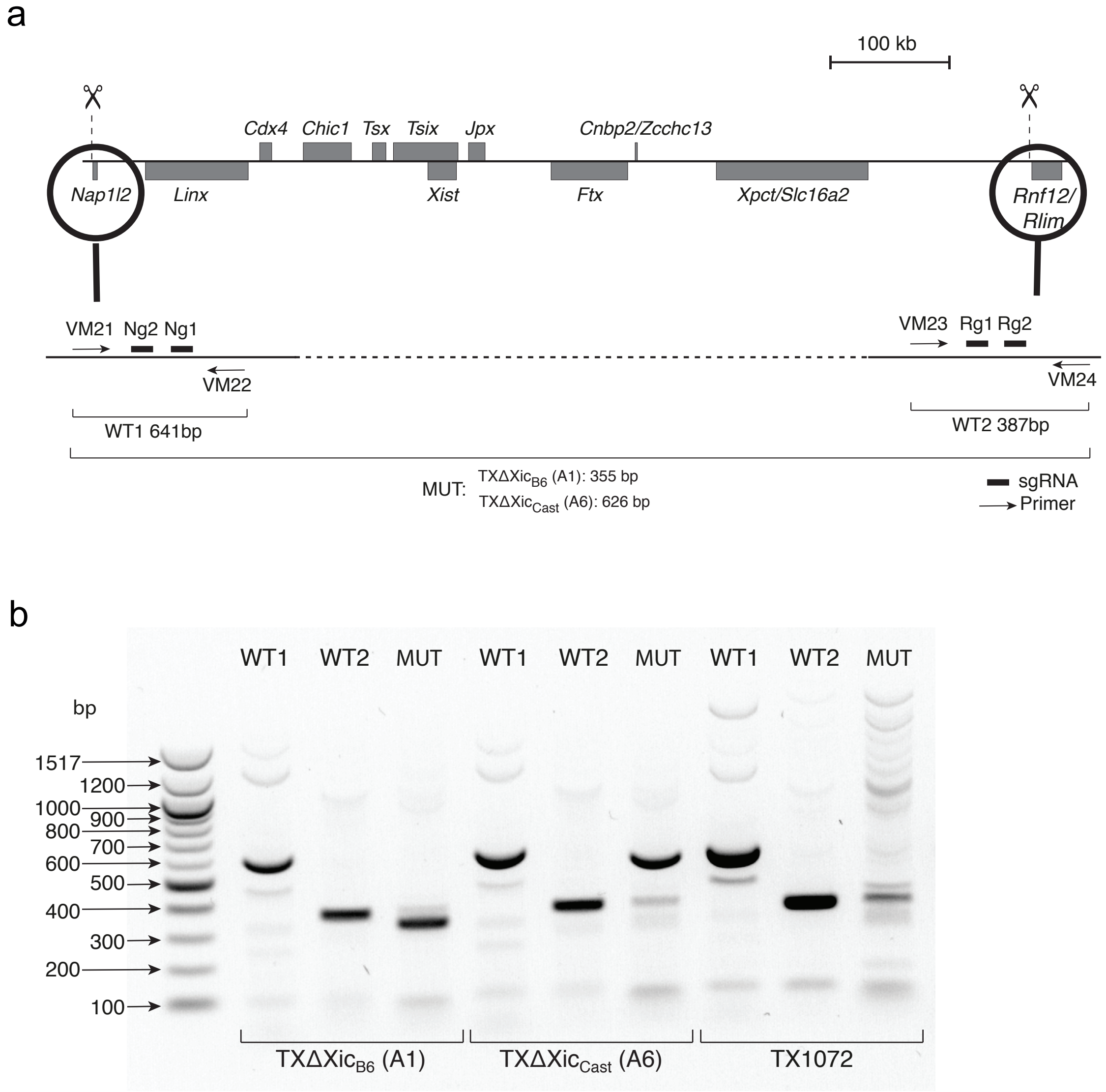

Supplementary Figure 10: Generation of Xic deletion mESC lines

(a) Schematic representation of the X inactivation center (top). Genes on the plus strand are shown above the line and genes on the minus strand below. Scissors demark the deleted region. The position of the sgRNA used to generate the deletion (bars) and of the primers (arrows) used for genotyping are shown together with the expected sizes for the PCR products. **(b)** Genotyping PCRs for clones A1 and A6 and the parental cell line TX1072. The experiment was performed twice with similar results and the identity of the PCR bands was confirmed by Sanger sequencing. Source data are provided as a Source Data file.

Supplementary Figure 11

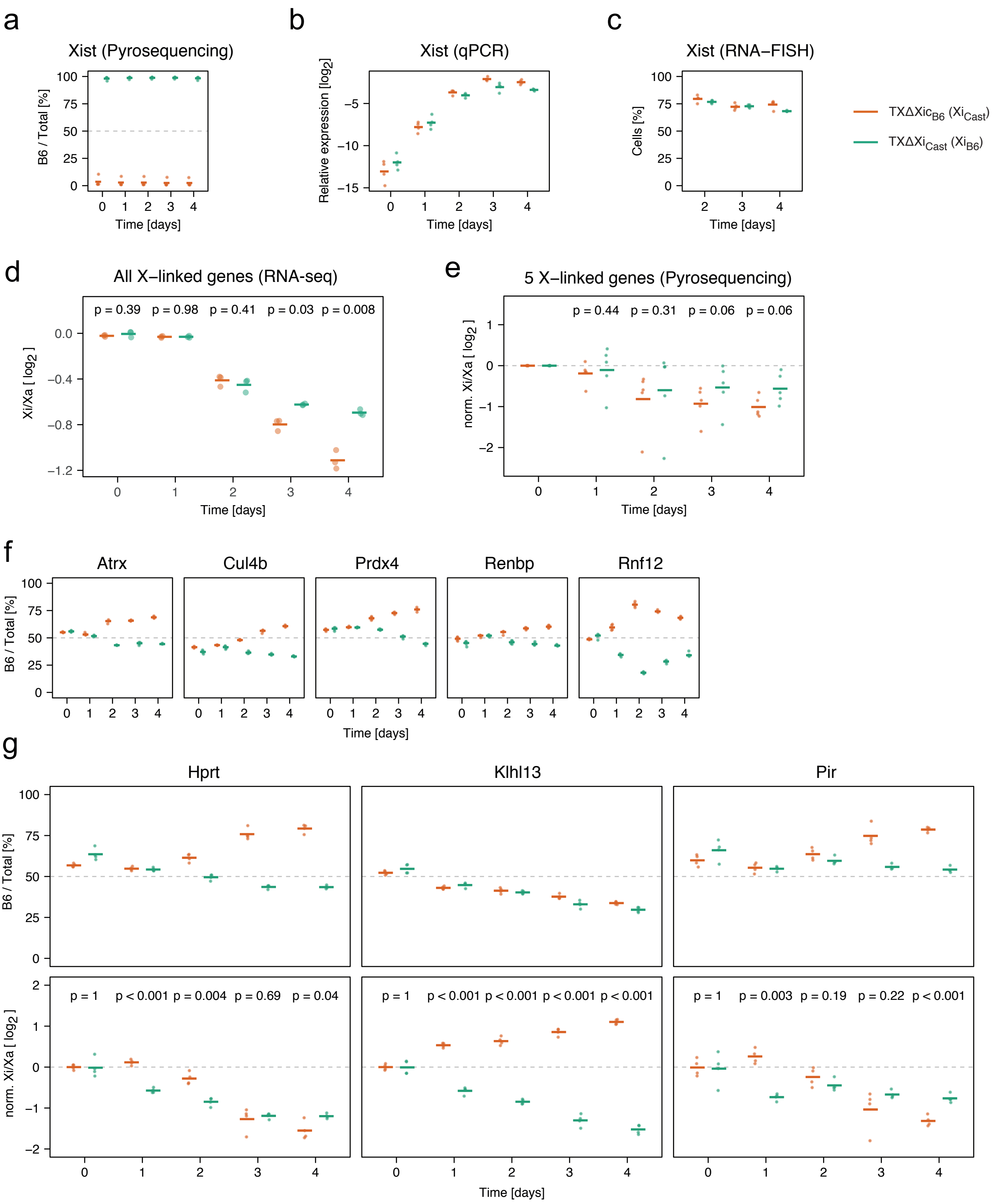

Supplementary Figure 11: Independent validation of differential silencing dynamics

(a-c) Comparison of Xist expression patterns in differentiating $TX\Delta Xic_{B6}$ and $TX\Delta Xic_{Cast}$ mESCs. Allelic expression of Xist was assessed by pyrosequencing (a), relative expression was assessed by qPCR (b) and the frequency of Xist upregulation by RNA-FISH (c) for the experiments shown in Fig. 7. Horizontal bars represent the mean of n=4 biological replicates, which are shown as dots. **(d)** Xi:Xa expression ratio assessed by bulk RNA-seq for all X-linked genes, estimated through summing up all X-chromosomal reads, except those mapping to the deleted region. Dots represent n=3 biological replicates, the horizontal bar the mean. Significance between the two cell lines according to an unpaired two-sided two-sample Student's T-test is indicated. **(e-f)** Allelic expression assessed by pyrosequencing for 5 genes that exhibited similar XP_{50} values on both alleles in the analysis in Fig. 6g (Renbp, Rlim, Prdx4, Cul4b, Atrx). In (e) the Xi:Xa expression ratios for 5 genes, averaged across 4 replicates and normalized to the average ratio on the pre-XCI state (day 0, dashed line), are shown (dots). The horizontal bar indicates the mean value across all n=5 genes. For each time point, the p-values were calculated using a Wilcoxon signed-rank two-sided test. In (f) dots represent n=4 biological replicates, horizontal lines indicate the mean values. **(g)** Allelic expression assessed by pyrosequencing for genes deemed as significantly differentially silenced between the two alleles in Fig. 6h. Dots represent n=4 biological replicates and horizontal lines the mean. The raw data (top) and the Xi:Xa expression ratios normalized to the average ratio on the pre-XCI state (day 0, dashed line) are shown (bottom). Additionally, p-values of a two-sample unpaired two-sided Student's T-test are shown. Source data are provided as a Source Data file.

Supplementary References

1. Hashimshony, T. et al. CEL-Seq2: sensitive highly-multiplexed single-cell RNA-Seq. Genome Biol. 17, 77 (2016).
