# Supplementary material for: Integrated analysis of Xist upregulation and X-chromosome inactivation with single-cell and single-allele resolution
Source: Nat Commun. 2021 Jun 15;12:3638. doi: 10.1038/s41467-021-23643-6 (PMC8206119; doi:10.1038/s41467-021-23643-6)
Supplement: Supplementary file 3 — Description of Additional Supplementary Files [file 41467_2021_23643_MOESM3_ESM.pdf]

Description of Additional Supplementary Files

Supplementary Data 1: Cell and gene filtering
Table summarizing the cell (Cell Filtering) and gene (Gene Filtering) filtering steps, as described in the Methods section. For both pre-processing steps, the table on top summarizes the number of cells or genes removed from the analysis for each filtering criterion.

Supplementary Data 2: Cell and gene classification
Classification of cells according to Xist expression, gene silencing and pseudotime and classification of X-linked genes according to their silencing behavior during XCI.

Supplementary Data 3: Identification of putative Xist regulators
Table summarizing the results of the differential expression analyses (*MAST: Xist High vs Low, MAST: ΔX High vs Low*) and the Spearman's correlation analyses (Spearman: Gene CPM vs Xist CPM, Spearman: Gene CPM vs *ΔX*), aiming to identify putative Xist and XCI regulators for each time point throughout cellular differentiation. In both analyses, the FDR column represents the Benjamini-Hochberg adjusted p-values.

Supplementary Data 4: Reagents
Primer and sgRNA sequences for cell line generation (TXdeltaXic), RNA FISH probes (FISH Probes), Pyrosequencing assays (Pyrosequencing), qPCR primers (qPCR) .
