# Supplementary material for: Integrated analysis of Xist upregulation and X-chromosome inactivation with single-cell and single-allele resolution
Source: Nat Commun. 2021 Jun 15;12:3638. doi: 10.1038/s41467-021-23643-6 (PMC8206119; doi:10.1038/s41467-021-23643-6)
Supplement: Supplementary file 8 — Reporting Summary [file 41467_2021_23643_MOESM8_ESM.pdf]

nature research

Corresponding author(s): Edda G Schulz, Annalisa Marsico

Last updated by author(s): Apr 2, 2021

Reporting Summary

Nature Research wishes to improve the reproducibility of the work that we publish. This form provides structure for consistency and transparency in reporting. For further information on Nature Research policies, see our Editorial Policies and the Editorial Policy Checklist.

Statistics

For all statistical analyses, confirm that the following items are present in the figure legend, table legend, main text, or Methods section.

n/a	Confirmed	
☐	☒	The exact sample size (*n*) for each experimental group/condition, given as a discrete number and unit of measurement
☒	☐	A statement on whether measurements were taken from distinct samples or whether the same sample was measured repeatedly
☐	☒	The statistical test(s) used AND whether they are one- or two-sided<br>*Only common tests should be described solely by name; describe more complex techniques in the Methods section.*
☐	☒	A description of all covariates tested
☐	☒	A description of any assumptions or corrections, such as tests of normality and adjustment for multiple comparisons
☐	☒	A full description of the statistical parameters including central tendency (e.g. means) or other basic estimates (e.g. regression coefficient) AND variation (e.g. standard deviation) or associated estimates of uncertainty (e.g. confidence intervals)
☐	☒	For null hypothesis testing, the test statistic (e.g. *F*, *t*, *r*) with confidence intervals, effect sizes, degrees of freedom and *P* value noted<br>*Give P values as exact values whenever suitable.*
☒	☐	For Bayesian analysis, information on the choice of priors and Markov chain Monte Carlo settings
☒	☐	For hierarchical and complex designs, identification of the appropriate level for tests and full reporting of outcomes
☐	☒	Estimates of effect sizes (e.g. Cohen's *d*, Pearson's *r*), indicating how they were calculated

Our web collection on statistics for biologists contains articles on many of the points above.

Software and code

Policy information about availability of computer code

Data collection	Zen v2.3 software (Zeiss) was used for aquisition and analysis of microscopy images.
Data analysis	Data processing was performed with Drop-seq (v.1.12), SNPsplit (v.0.3.2), STAR (v.2.5.2b), and R (v.3.6.1) using the R packages data.table (v.1.12.8), doParallel (v.1.0.15), foreach (v.1.5.0), edgeR (v.3.26.8), plyr (v.1.8.6), Rsubread (v.1.34.7), R.utils (v.2.10.1). All downstream analyses were performed using custom R scripts, making use of a the open-source R packages biomaRt (v.2.40.5), data.table (v.1.12.8), doParallel (v.1.0.15), dplyr (v.1.0), edgeR (v.3.26.8), ggalluvial (v.0.11.3),ggplot2 (v.3.3.2), ggrepel (v.0.8.2), gridExtra (v.2.3), lsr (v.0.5), MAST (v.1.10), matrixStats (v.0.56.0), monocle (v.2.12.0), multidplyr (v.0.0.0.9000), openxlsx (v.4.2.2), pcaMethods (v.1.76.0), pheatmap (v.1.0.12), plyr (v.1.8.6), readxl (v.1.3.1), reshape2 (v.1.4.4), R.utils (v.2.10.1), scran (v.1.12.1), tidyr (v.1.1.0), umap (v.0.2.6.0), UpSetR (v.1.4.0), velocyto.R (v.0.6). The code is available on https://github.com/EddaSchulz/Pacini_paper.

For manuscripts utilizing custom algorithms or software that are central to the research but not yet described in published literature, software must be made available to editors and reviewers. We strongly encourage code deposition in a community repository (e.g. GitHub). See the Nature Research guidelines for submitting code & software for further information.

Data

Policy information about availability of data

All manuscripts must include a data availability statement. This statement should provide the following information, where applicable:

- Accession codes, unique identifiers, or web links for publicly available datasets
- A list of figures that have associated raw data
- A description of any restrictions on data availability

ScRNA-seq and bulk RNA-seq data generated during this study are available via GEO with identifier GSE151009 [https://www.ncbi.nlm.nih.gov/geo/query/acc.cgi?acc=GSE151009] as raw fastq files and as the unfiltered not-AS and AS count tables together with the list of SNPs used for allele-specific analysis. Source data for all

figures are provided with this paper.

Field-specific reporting

Please select the one below that is the best fit for your research. If you are not sure, read the appropriate sections before making your selection.

- [x] Life sciences
- [ ] Behavioural & social sciences
- [ ] Ecological, evolutionary & environmental sciences

For a reference copy of the document with all sections, see nature.com/documents/nr-reporting-summary-flat.pdf

Life sciences study design

All studies must disclose on these points even when the disclosure is negative.

Sample size	No statistical methods were used to determine sample size. The sample size (400 cells per time point) was chosen, because this is the minimum possible with the microfluidics systems used. Smaller experiments were performed in 4 independent biological replicates, since that number is feasible and allows statistical analysis of the results.
Data exclusions	A series of filtering steps were performed to exclude low-quality cells and lowly expressed genes from the analysis. The exclusion criteria were not pre-established. Information on mitochondrial reads, ERCC spike-in reads and detected genes were used, because they are known to be associated with cell viability, and it is state-of-the-art to use them for cell filtering.
Replication	Due to the high costs associated with the experiment it was not repeated.
Randomization	A random subset of cells was captured at the microfluidic chamber at each time point. For all other experiments, ES cells used in this study were grown under identical conditions and differentiation was induced at different days for each biological replicate. No randomization was used.
Blinding	Cells were grown under identical conditions and subsequently all samples are processed in parallel, such that blinding is not applicable.

Reporting for specific materials, systems and methods

We require information from authors about some types of materials, experimental systems and methods used in many studies. Here, indicate whether each material, system or method listed is relevant to your study. If you are not sure if a list item applies to your research, read the appropriate section before selecting a response.

Materials & experimental systems

n/a	Involved in the study	
x		Antibodies
	x	Eukaryotic cell lines
x		Palaeontology and archaeology
x		Animals and other organisms
x		Human research participants
x		Clinical data
x		Dual use research of concern

Methods

n/a	Involved in the study	
x		ChIP-seq
x		Flow cytometry
x		MRI-based neuroimaging

Eukaryotic cell lines

Policy information about cell lines

Cell line source(s)	Mouse embryonic stem cells derived from a blastocyst.
Authentication	No authentication required because the cell lines was derived in the laboratory. Number of X-chromosomes present was tested regularly.
Mycoplasma contamination	Cell line was tested for mycoplasma regularly, with negative results.
Commonly misidentified lines (See ICLAC register)	no such cell line used.
